# Large differencies in age-specific survival in multiple myeloma in the nordic countries

**DOI:** 10.1038/s41408-024-01026-6

**Published:** 2024-03-11

**Authors:** Kari Hemminki, Frantisek Zitricky, Asta Försti, Raija Silvennoinen, Annette Vangsted, Markus Hansson

**Affiliations:** 1 Biomedical Center, Faculty of Medicine and Biomedical Center in Pilsen, Charles University in Prague, 30605 Pilsen, Czech Republic; 2 Division of Cancer Epidemiology, German Cancer Research Center (DKFZ), Im Neuenheimer Feld 580, D-69120 Heidelberg, Germany; 3 Hopp Children’s Cancer Center (KiTZ), Heidelberg, Germany; 4 Division of Pediatric Neurooncology, German Cancer Research Center (DKFZ), German Cancer Consortium (DKTK), Heidelberg, Germany; 5 Department of Hematology, Helsinki University Hospital Comprehensive Cancer Center, & University of Helsinki, Helsinki, Finland; 6 Department of Hematology, Rigshospitalet, Copenhagen, Denmark; 7 Department of Internal Medicine and Clinical Nutrition, Sahlgrenska University Hospital/Sahlgrenska Academy, Bruna stråket 5 plan 5, 41325 Göteborg, Sweden; 8 Hematology and Transfusion Medicine, Department of Laboratory Medicine, BMC B13, 221 84 Lund, Sweden

**Keywords:** Translational research, Cancer epidemiology

Dear Editor,

Treatment of multiple myeloma (MM), like most hematological neoplasms, has developed fast and completely novel therapeutic modalities have been introduced [1]. In the late 1980s autologous stem cell transplantation (ASCT) was introduced in hematology, and for MM it was used in combination with high-dose melphalan treatment [2]. Eligibility for ASCT was considered by fitness of the patients and their age (<65 years), which however has been increased to 70 or 75 years [3]. In the Nordic countries some 30% of MM patients undergo this primary treatment [4, 5]. Vincristine, adriamycin, and dexamethasone were introduced as induction treatment. This was followed by about 2010 by agents based on novel mechanisms of action, including immunomodulatory agents (e.g., thalidomide, lenalidomide) and proteasome inhibitors (e.g., bortezomib), which transformed the management of MM [4–6]. Novel proteasome inhibitors and immunomodulatory agents have been introduced, and most recently immunotherapies have become available. Treatment of patients not eligible for ASCT diagnosed at age >75 years was compared in Sweden and Denmark up to year 2020 and found to be approximately similar [6]. Even though the disease was more advanced with higher ISS stage and with higher degree of renal impairment in the old patients, they responded and tolerated treatment.

Survival has improved in MM in Sweden and USA but survival in elderly patients has remained below that of younger patients [7–9]. As treatments, such as ASCT and extensive chemotherapeutic regiments, may be limited to or tolerated by the fit and young patients it is relevant to investigate the up-to date age-group specific survival which was recently enabled in the NORDCAN database, allowing an analysis through a half century up to year 2021 [10]. These results for 1-year and 5-year relative survival are presented here for Denmark (DK), Finland (FI), Norway (NO) and Sweden (SE), with methods shown in *Supplement*, and reasons for the country-specific differences are discussed.

The NORDCAN database included 8660 male MM patients in DK, 7251 in FI, 9249 in NO and 16,212 in SE. The related female patient numbers were 7091, 7817, 7598 and 13,172. In the first 10-year period the oldest patients (80−89 years) accounted for 10–15% of all male patients and 15−20% of female patients. In the last 10 years, the oldest male and DK female patients accounted for 21% of all patients and the other oldest female patients accounted for 28% of all patients.

We plotted 1- and 5-year relative survival curved by age-groups for the largest country, SE (Fig. 1). Survival developed well through the 50 years; for 1-year survival even 80−89-year-old patient approached 80% survival, and were able to narrow the gap to the younger patients. Improvement in 5-year survival was almost linear for patients below 60 year of age but for the older patients hardly any improvement took place until about year 2000 when a steep catch-up towards the younger patients.

**Fig. 1:**
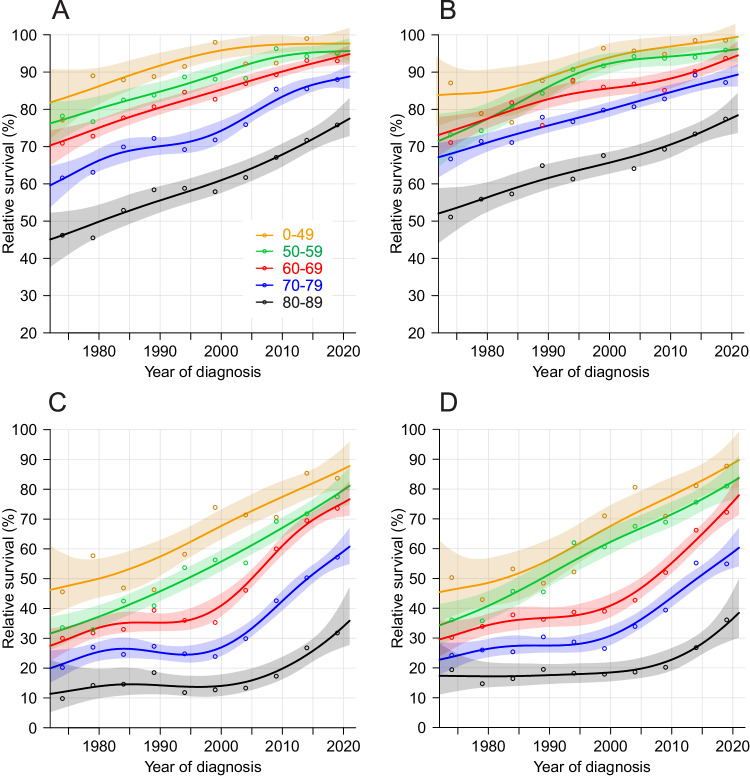
Relative age-group specific 1- and 5-year survival with 95%CIs in Swedish men and women from 1972−76 to 2017−21. Top panels are for 1-year survival in men (**A**) and women (**B**); the bottom ones are for 5-year survival in men (**C**) and women (**D**).

Based on results from Supplementary Table 1 on overall survival trends in MM we selected DK (largest survival increase) and FI (smallest increase) for detailed plotting of 1-year survival curves (Supplementary Fig. 1). DK survival increased in all age groups, and the large differences in the first period somewhat narrowed over time. The initial survival in FI was better than that in DK but in older patients survival improvements were slow and the 80–89-year-old patients reached survival of 60%, some 20% units below their DK mates.

Initial 5-year age-specific survival developed poorly in DK until 1995 when a strong boost increased survival first for younger patients when ASCT was introduced in 1994, followed by the elderly (Supplementary Fig. 2). Importantly, survival of even the oldest men and women was over 50%. In FI survival was initially favorable but towards the end survival was below the DK figures; the 80–89-year-old patients reached only a 20% 5-year survival.

We compared the first (1972−76) and the last (2017−21) 5-year survival figures in the Nordic counties in Table 1. Survival improvements were higher for the patients diagnosed before age 50 years compared to the 80−89-year old patients (except in NO men). As a notable contrast to the other countries, the 50-year survival gain in young DK patients was 57% units and in the oldest patients it was 41% units. NO and SE young patients enjoyed a survival gain of 30−40% units and the oldest patients of about 20% units, but no gain for old FI patients and only 10% units for old NO women.

**Table 1:** 5-year relative survival % in multiple myeloma in the Nordic countries between the periods 1972–1976 and 2017–2021, among the youngest and oldest age groups.

MALE 5-YEAR SURVIVAL AMONG 0-49 YEAR OLD					FEMALE 5-YEAR SURVIVAL AMONG 0-49 YEAR OLD			
Period	Denmark	Finland	Norway	Sweden	Denmark	Finland	Norway	Sweden
1972–1976	27.1 [14.4–36.8]^a^	42.4 [30.3–59–4]	60.9 [45.4–79.8]	45.6 [34.0–61.0]	36.9 [26.4–51.6]	41.1 [27.1–62.5]	43.4 [33.7–55.8]*	50.3 [37.5–67.4]
2017–2021	84.1 [75.0–94.2]	77.3 [65.2–91.6]	84.6 [76.9–95.1]	83.7 [76.7–91.5]	90.6 [82.6–99.4]	73.5 [65.2–82.7]^a^	86.0 [76.6–96.2]	87.7 [79.8–96.4]
Improvement^b^	57.0	34.9	23.7	38.1	53.7	32.4	42.6	37.4

MALE 5-YEAR SURVIVAL AMONG 80–89 YEAR OLD					FEMALE 5-YEAR SURVIVAL AMONG 80–89 YEAR OLD			
Period	Denmark	Finland	Norway	Sweden	Denmark	Finland	Norway	Sweden
1972–1976	11.6 [3.0–44.3]	21.6 [13.7–34.3]^c^	22.4 [16.4–30.8]^c^	9.8 [4.2–22.9]	10.8 [5.7–20.4]^c^	17.2 [7.3–40.8]	20.4 [9.8–41.3]	19.4 [12.0–31.2]
2017–2021	52.5 [41.9–65.8]	14.3 [9.2–22.4]	47.0 [37.4–59.1]	31.8 [25.6–39.6]	53.1 [43.1–65.5]	20.6 [15.4–27.7]	30.2 [22.7–40.0]	36.1 [29.5–44.2]
Improvement^b^	40.9	−7.3	24.6	22	42.1	3.4	9.8	16.7

*Significant difference (non-overlapping 95% CIs).

^a^Age-group 50−59 years, as younger cases were few.

^b^Difference between the two periods in % units.

^c^Age group 70−79 years, as older cases were few.

Survival is dependent on stage at diagnosis and we collected ISS stage distributions from the available sources (*Supplement*, no data for NO were available). DK stage distribution was more favorable than that of SE, which was more favorable than that of FI (Supplementary Table 2).

Survival in MM in the western countries has generally developed well and the present results confirm the positive trend. The main factors behind the development are thought to be improved medication, diagnostics, control of infections and supportive care, as reviewed [1]. The present results deliver three messages. The first one is that old MM patients are a disadvantaged group, and none of the present countries were able to convincingly narrow the 5-year survival gab between the youngest and oldest patients. While some previous studies have shown data on the age bias, some of them are old in view of the current development, some cover broad age ranges and some, like our earlier study, only cover a single country [7, 9, 11]. From Supplementary Fig. 1 and 2 we could see that the age disadvantage struck already in 1-year survival. During the first year, 40% of the FI 80−89-year old MM patients had died and only 20% of the patients remained alive in 5 years. As seen in the DK data, the prerequisite for old patients’ long-term survival was that a large proportion survive year 1 (80%), paving the prospect for surviving next 4 years (> 50%).

The second message is that neighboring countries with historical ties in medicine and with a joint Nordic Myeloma Study Group since year 1987 show different survival profiles in their MM patients. One needs to point out that the Nordic cancer registries have collaborated longer than the current study duration of 50 years [12]. It is thus likely that the survival figures are accurate and comparable. Data from DK and SE show that the national treatment guidelines are rapidly implemented in practice [5]. The available ISS stage distribution gave an important clue about reasons for the survival differences between the countries; stages were most favorable in DK and least favorable in FI. DK established a national cancer program in year 2000 in response to poor survival data in international comparisons [13]. It guaranteed funding for infrastructure, centralized treatment and instituted facilitated pathways for treatment and patient review by multidisciplinary expert teams. NO and SE followed the model afterwards but FI is still considering. In DK progress in treatment of MM was contributed by the Danish Myeloma study group from 2005, the national quality data base on MM and annual updating of guidelines together with a strong patient organization [14]. FI has been slowly recovering from the deep economic crisis in the 1990s (doi: 10.1787/8643de7e-en) which impeded health care reforms, such as facilitated patient pathways from primary to secondary care. As the consequence of economic restrictions, availability of novel drugs to hematology clinics has been delayed [15]. The positive side of the close collaboration between the neighboring countries is to learn from each other and the Nordic Myeloma Study Group could spearhead this effort of mutual assistance.

The final message is that survival in MM can increase only marginally unless the oldest patients are enrolled into long-term positive survival trajectories. In 2012-22, the 80−89-year-old population was about 22% of male and 27% of female patients and, by all account, the proportion of old patients will continue to increase. In the Nordic setting, DK was able to increase 5-year survival fivefold for the 80−89-year-old patients giving hope that this may be doable also elsewhere.

The main limitation of the study is that NORDCAN contains no individual data nor even grouped data on clinical presentation or treatment. An unanswered question is if the patient population has remained homogeneous over the long observation time. We noted that the proportion of 80−89-year-old patients had increased markedly. This may be related to the aging of the population but increased diagnostic activity in the old population may have also contributed to the increase, as has been suggested [5]. Another limitation is the generalizability of the results, originating from the four Nordic countries with over 25 million inhabitants. Treatment may be further modified and more advanced in other settings (and certainly varies within any individual Nordic country). However these are the most up-to-date survival figures for the entire population.

The strong DK 5-year survival improvement started at around 1995 and it is likely that various therapeutic novelties in combination with diagnostic and care improvements individually contributed to the survival gains. Accomplishing this in 20 years and making sure that the elderly patients were kept on with the positive development was a remarkable achievement and encouragement to other countries. The challenge of the old patients is not over yet even in DK, but it was encouraging to note that the survival curves for the 80−89-year old patients kept the steep upward trend up to year 2021. Among the drugs that have recently been introduced for MM, the CD-38 blocking antibodies can be used for old patients and those failing other therapies.

### Supplementary information

LARGE DIFFERENCIES IN AGE-SPECIFIC SURVIVAL IN MULTIPLE MYELOMA IN THE NORDIC COUNTRIES

## Data Availability

A public database was used as the source of data.
