# Supplementary material for: Large differencies in age-specific survival in multiple myeloma in the nordic countries
Source: Blood Cancer J. 2024 Mar 11;14(1):43. doi: 10.1038/s41408-024-01026-6 (PMC10928156; doi:10.1038/s41408-024-01026-6)
Supplement: Supplementary file 1 — LARGE DIFFERENCIES IN AGE-SPECIFIC SURVIVAL IN MULTIPLE MYELOMA IN THE NORDIC COUNTRIES [file 41408_2024_1026_MOESM1_ESM.docx]

SUPPLEMENT

LARGE DIFFERENCIES IN AGE-SPECIFIC SURVIVAL IN MULTIPLE MYELOMA IN THE NORDIC COUNTRIES

Kari Hemminki^1,2^, Frantisek Zitricky^1^, Asta Försti^3,4^, Raija Silvennoinen^5^, Annette Vangsted^6^, Markus Hansson^7,8^

Previous survival data in multiple myeloma (MM) have shown large improvements but old patients have been disadvantaged. The novel NORDCAN age-specific relative survival data allowed us to examine most recent age-group specific survival between 1972 and 2021 in Denmark (DK), Finland (FI), Norway (NO) and Sweden (SE).

METHODS

The data were obtained from the NORDCAN database 2.0 which has been created through collaboration between the Nordic cancer registries (1-3). The database was accessed at the International Agency for Cancer (IARC) website (<https://nordcan.iarc.fr/en>) in fall of 2023 (3). Using this resource, we extracted data on overall and age-group specific 1-year and 5-year relative survival and relevant case numbers from 1972 until the end of 2021. A significant difference in survival between the consecutive 5-year follow-up periods was considered when 95% confidence intervals (CIs) between the two survivals were non-overlapping.

The temporal trends in relative survival were modelled using Bayesian generalized additive models (with Gaussian link function) (4). The modelling was performed on cumulative hazard scale, which allowed to model input uncertainty for the asymmetric CIs provided by NORDCAN.

Stage distribution (International Staging System, ISS) of MM was analyzed based on data from the national MM registers of DK (4691 patients from years 2005 to 2018) and of SE (7334 patients from years 2008 to 2019) (5). The national register in FI did not collect ISS data. Instead data were obtained from the hospital district of Helsinki and the surrounding Uusimaa, which is the most populous of the five FI hospital districts (6). MM patients (509) were diagnosed between 2013 and 2019 and they included 161 ASCT- treated and 348 patients not treated with ASCT but who received immunochemotherapy. Of these patients 160 were diagnosed at age below 65 years, and of the 249 older patients 155 were diagnosed at ages over 74 years (6). We note that an unknown number of diagnosed MM patients were not included in the study and the data are not fully comparable to the DK and SE register data.

RESULTS

Overall relative 1- and 5-year survival for men and women in MM is shown in **Supplementary Table 1**. 1-year survival increased from about 60% in men and 65% in women (1972-1976) to 90% (2017-2021), except for FI men (81.3%) and women (85.2%). Asterisks in Table 1 show significant increases between the consecutive 5-year periods, all of which took place at year 2000 or later. The increases in 5-year survival were also large, from about 30% (lower in DK) to 70% in DK, 65% in NO and SE, and 50% in FI men. The DK survival increases were large after year 2000, ca. 30 % units.

Based on results from **Table 1** we selected DK (largest survival increase) and FI (smallest increase) data for detailed plotting of 1-year survival curves (**Supplementary Fig. 1**). DK survival increased in all age groups, and the large differences in the first period somewhat narrowed over time. Survival in patients diagnosed below 70 years was at least 90% and the oldest male and female patients reach a survival of 80%. The initial survival in FI was better than that in DK. Patients younger than 60 years reached a survival of over 90% but in older patients survival improvements were slow and the 80-89-year-old patients reached survival of 60%, some 20 % units below their DK mates.

Extending scrutiny to 5-year age-specific survival in DK and FI revealed a different course of development (**Supplementary Fig. 2**). DK survival developed poorly until 1995 when a strong boost increased survival first for younger patients when ASCT was introduced in 1994, followed by the elderly. DK women diagnosed below age 60 years reached a 5-year survival of 90% while men were less than 10 % units behind. Importantly, survival of even the oldest men and women was over 50%. In FI survival in the two youngest age groups was very favorable initially but towards the end survival in any age group was some 20 % units below the DK figures, and 30 % units for the 80-89-year-old patients who reached only a 20% 5-year survival.

We tried to explain the noted national survival differences by the stage at diagnosis, using the national MM register data for DK and SE, and regional (selected) data for FI. ISS stage distribution differed between the countries (**Supplementary Table 2**). DK patients were diagnosed at significantly lower stages than SE patients, which were diagnosed at significantly lower stages than FI patients. A concern about the regional FI data is that the patients were selected based on treatment and an unknown number of patients were not included. It is likely that the excluded group of non-treated patients were of relatively high stage which would further weight the FI stage distribution towards higher stage.

CONCLUSIONS

We summarized nation-wide MM survival tends from DK, FI, NO and SE through a half century up to 2021. In 2017-2021 5-year survival for patients diagnosed before age 50 years varied between 73 and 90%, depending on the country; for patients aged 80-89 years survival varied between 30 and 47% in NO and SE but was over 50% for Danes. Even young Finns survived worse than their Nordic mates but for 80-89-year-old Finns survival was 20% or less, and it had not improved over time. The results on stage distribution may be an important explanation to the positive survival data in DK and to the slow improvement in FI. The early diagnosis in DK may be in part a result of the national cancer policy from year 2000 emphasizing facilitated patient pathways (7). According to the literature older patients with advanced disease may tolerate treatment and respond well. The DK success in the disadvantaged old population is encouraging news to the other countries. Even the old patients may benefit from an active treatment.

LEGENDS TO FIGURES

Fig. 1. Relative age-group specific 1-year survival with 95%CI in Danish men and women (top) and Finnish men and women in 5-year periods from 1972-76 to 2017-21. Some curves for the youngest and oldest patients were truncated because of less than 30 patients at the beginning of follow-up.

Fig. 2. Relative age-group specific 5-year survival with 95%CI in Danish men and women (top) and Finnish men and women in 5-year periods from 1972-76 to 2017-21. Some curves for the youngest and oldest patients were truncated because of less than 30 patients at the beginning of follow-up.

REFERENCES

1. Engholm G, Ferlay J, Christensen N, Bray F, Gjerstorff ML, Klint A, et al. NORDCAN--a Nordic tool for cancer information, planning, quality control and research. Acta Oncol. 2010;49(5):725-36.

2. Pukkala E, Engholm G, Hojsgaard Schmidt LK, Storm H, Khan S, Lambe M, et al. Nordic Cancer Registries - an overview of their procedures and data comparability. Acta Oncol. 2018;57:440-55.

3. Larønningen S AG, Bray F, Engholm G, Ervik M, Guðmundsdóttir EM, Gulbrandsen J, Hansen HL, Hansen HM, Johannesen TB, Kristensen S, Kristiansen MF, Lam F, Laversanne M, Miettinen J, Mørch LS, Ólafsdóttir E, Pejicic S, Petterson D, Steig BÁ, Skog A, Tian H, Aagnes B, Storm HH. NORDCAN: Cancer Incidence, Mortality, Prevalence and Survival in the Nordic Countries, Version 9.3 (02.10.2023). 2023.

4. Tichanek F, Försti A, Liska V, Hemminki A, Hemminki K. Survival in Colon, Rectal and Small Intestinal Cancers in the Nordic Countries through a Half Century. Cancers. 2023;15(3).

5. Moore KLF, Turesson I, Genell A, Klausen TW, Knut-Bojanowska D, Redder L, et al. Improved survival in myeloma patients-a nationwide registry study of 4,647 patients ≥75 years treated in Denmark and Sweden. Haematologica. 2023;108(6):1640-51.

6. Vikkula J, Uusi-Rauva K, Ranki T, Toppila I, Aalto-Setälä M, Pousar K, et al. Real-world evidence of multiple myeloma treated from 2013 to 2019 in the Hospital District of Helsinki and Uusimaa, Finland. Future Oncol. 2023;19(30):2029-43.

7. Probst HB, Hussain ZB, Andersen O. Cancer patient pathways in Denmark as a joint effort between bureaucrats, health professionals and politicians--a national Danish project. Health Policy. 2012;105(1):65-70.

| **SUPPLEMENTARY TABLE 1. 1- and 5-year relative survival in multiple myeloma in the Nordic countries between 1972 and 2021** | | | | | | | | | |
| --- | --- | --- | --- | --- | --- | --- | --- | --- | --- |
|  |  |  |  |  |  |  |  |  |  |
| MALE 1-YEAR SURVIVAL | |  |  |  |  | FEMALE 1-YEAR SURVIVAL | |  |  |
| Period | Denmark | Finland | Norway | Sweden |  | Denmark | Finland | Norway | Sweden |
| 1972-1976 | 55.1[49.9-60.8] | 59.0[53.9-64.5] | 66.3[62.5-70.4] | 66.5[63.6-69.5] |  | 59.9[54.5-65.8] | 63.6[59.2-68.4] | 72.4[68.6-76.5] | 69.1[66.2-72.2] |
| 1977-1981 | 56.9[52.7-61.6] | 68.9[64.1-74.0] | 67.4[64.0-70.9] | 68.5[65.8-71.4] |  | 60.2[55.5-65.3] | 71.7[67.9-75.6] | 73.2[69.3-77.3] | 72.5[69.4-75.7] |
| 1982-1986 | 58.5[54.6-62.6] | 72.3[68.4-76.4] | 72.0[68.7-75.3] | 73.8[71.3-76.3] |  | 60.9[56.9-65.2] | 74.1[70.6-77.7] | 76.4[72.8-80.0] | 74.2[71.4-77.1] |
| 1987-1991 | 60.2[56.5-64.2] | 68.1[64.4-72.0] | 70.0[66.7-73.5] | 76.4[74.1-78.8] |  | 66.0[62.2-70.0] | 72.9[69.7-76.3] | 76.5[72.9-80.2] | 77.6[75.1-80.3] |
| 1992-1996 | 66.4[62.9-70.2] | 71.2[67.6-74.9] | 74.5[71.3-77.9] | 77.8[75.7-80.0] |  | 73.4[69.8-77.1] | 71.3[68.2-74.6] | 74.9[71.1-78.9] | 81.1[78.8-83.4] |
| 1997-2001 | 67.9[64.3-71.7] | 75.1[71.9-78.3] | 75.9[72.9-79.1] | 78.6[76.5-80.7] |  | 72.7[69.2-76.4] | 75.3[72.4-78.2] | 78.6[75.5-81.9] | 83.7[81.6-85.8] |
| 2002-2006 | 76.3[73.3-79.4]* | 76.0[73.0-79.2] | 78.0[75.3-80.9] | 80.8[78.8-82.9] |  | 78.0[74.8-81.3] | 77.8[75.0-80.7] | 80.9[78.1-83.8] | 84.0[82.0-86.0] |
| 2007-2011 | 81.2[78.7-83.8] | 78.1[75.2-81.0] | 81.1[78.7-83.6] | 86.4[84.7-88.1]* |  | 82.4[79.7-85.2] | 80.3[77.5-83.2] | 85.4[83.0-87.9] | 84.7[82.8-86.6] |
| 2012-2016 | 85.8[83.9-87.8]* | 81.5[79.1-84.1] | 85.5[83.3-87.7] | 88.6[87.2-90.0] |  | 88.7[86.7-90.8]* | 85.2[83.1-87.3] | 85.3[83.0-87.7] | 89.0[87.4-90.7]* |
| 2017-2021 | 90.9[89.3-92.6]* | 81.3[78.9-83.7] | 87.0[85.2-88.8] | 89.6[88.2-90.9] |  | 90.9[89.1-92.7] | 85.2[83.0-87.5] | 90.3[88.5-92.2]* | 90.3[88.9-91.8] |
| MALE 5-YEAR SURVIVAL | |  |  |  |  | FEMALE 5-YEAR SURVIVAL | |  |  |
| Period | Denmark | Finland | Norway | Sweden |  | Denmark | Finland | Norway | Sweden |
| 1972-1976 | 17.5[13.3-23.0] | - | 29.3[25.1-34.1] | 26.6[23.7-30.0] |  | - | 26.9[22.6-32.1] | 29.5[25.2-34.6] | 30.2[27.0-33.9] |
| 1977-1981 | 16.6[13.3-20.8] | 30.9[26.0-36.8] | 29.3[25.7-33.3] | 31.1[28.0-34.5] |  | 22.5[18.2-27.9] | 30.7[26.6-35.3] | 33.2[28.9-38.2] | 30.1[26.8-33.9] |
| 1982-1986 | 21.6[17.9-26.0] | 33.5[29.1-38.6] | 28.6[24.9-32.9] | 31.1[28.3-34.2] |  | 19.9[16.4-24.3] | 34.8[30.8-39.4] | 30.7[26.6-35.3] | 34.2[31.0-37.7] |
| 1987-1991 | 22.6[19.0-26.9] | 30.9[27.0-35.5] | 32.2[28.2-36.6] | 33.8[31.0-36.9] |  | 25.4[21.6-29.9] | 31.4[27.5-35.8] | 32.1[28.0-36.8] | 35.1[32.0-38.6] |
| 1992-1996 | 24.2[20.8-28.2] | 32.3[28.4-36.8] | 32.3[28.6-36.4] | 34.8[32.1-37.7] |  | 27.5[23.7-32.0] | 34.3[30.7-38.4] | 32.6[28.3-37.5] | 38.3[35.2-41.6] |
| 1997-2001 | 29.7[26.2-33.7] | 39.2[35.5-43.2] | 38.9[35.2-43.0] | 36.8[34.2-39.6] |  | 33.3[29.6-37.5] | 35.9[32.5-39.7] | 39.6[35.7-44.0] | 39.7[36.8-42.9] |
| 2002-2006 | 40.0[36.3-44.0]* | 41.9[38.3-45.9] | 40.6[37.2-44.3] | 41.1[38.5-43.9] |  | 45.7[41.7-50.0]* | 44.1[40.4-48.1]* | 41.9[38.1-46.0] | 45.3[42.4-48.4] |
| 2007-2011 | 51.5[48.0-55.3]* | 44.9[41.5-48.7] | 46.6[43.4-50.0] | 51.4[48.9-54.0]* |  | 50.8[47.1-54.9] | 45.9[42.3-49.8] | 52.8[49.2-56.6]* | 48.7[46.0-51.7] |
| 2012-2016 | 61.4[58.4-64.6]* | 47.1[43.7-50.8] | 59.3[56.1-62.7]* | 59.8[57.5-62.3]* |  | 68.6[65.4-72.1]* | 55.5[52.3-58.8]* | 56.7[53.3-60.3] | 60.5[57.8-63.3]* |
| 2017-2021 | 69.0[65.9-72.3]* | 50.0[46.7-53.6] | 65.9[62.8-69.1]* | 64.4[62.2-66.8] |  | 73.2[70.1-76.5] | 56.4[53.2-59.8] | 64.1[60.9-67.6]* | 65.1[62.5-67.8] |
|  |  |  |  |  |  |  |  |  |  |
| * Non-overlapping 95%CIs with the previous survival figure. | | | | |  |  |  |  |  |

**Supplementary Table 2.** International staging system distribution in MM in Denmark, Sweden and Finland

| ISS stage | Denmark  N (%) | Sweden  N (%) | Finland  N (%) |
| --- | --- | --- | --- |
| ISS stage I | 925 (23.9) | 1051 (20.3) | 67 (15.3) |
| ISS stage II | 1447 (37.4) | 2256 (43.6) | 213 (48.5) |
| ISS stage III | 1494 (38.6) | 1864 (36.0) | 159 (36.2) |

According to Pearson's chi-squared test, there is a significant difference in the distributions between DK and SE (p<0.001), between DK and FI (p<0.001) and between SE and FI (p=0.02).


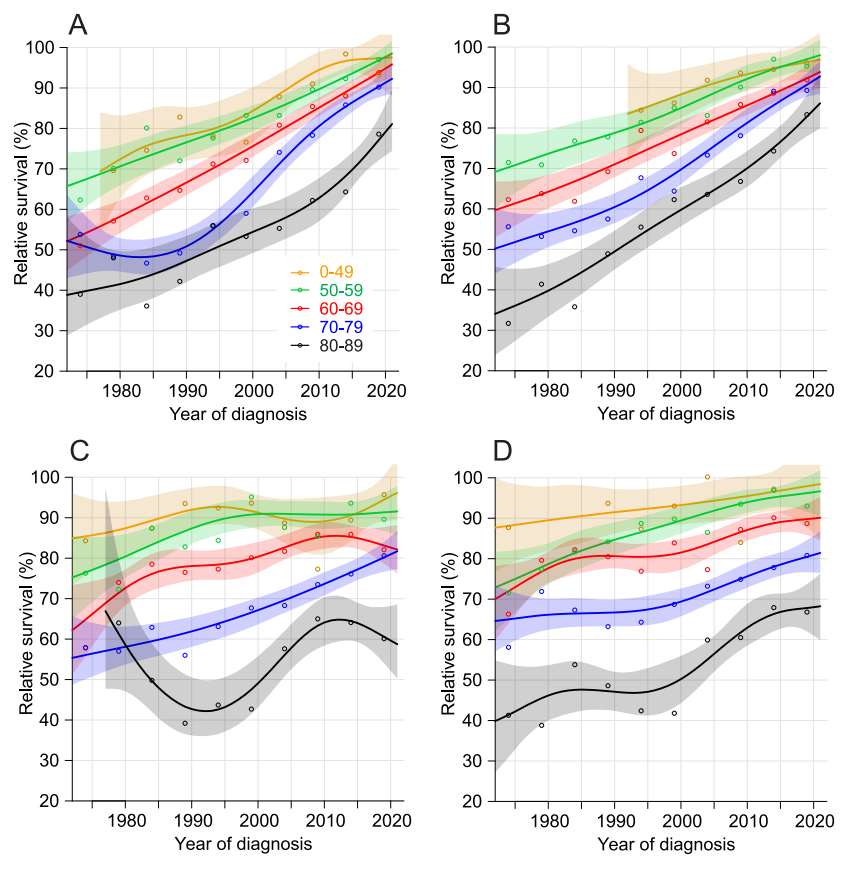


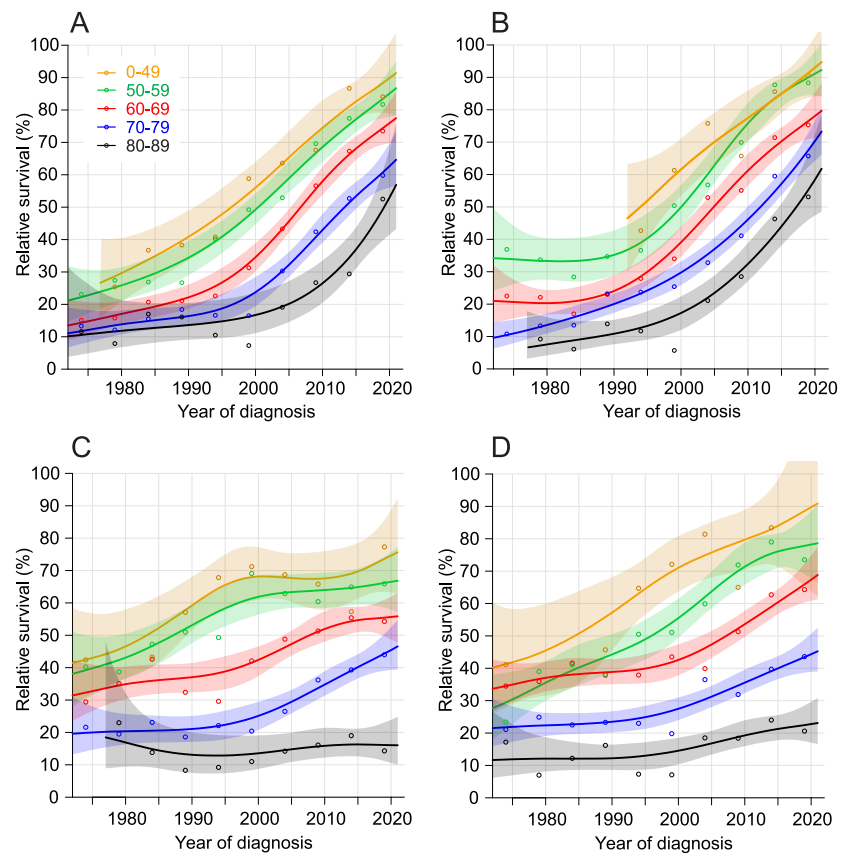


Suppl. Fig 2
